# Trends in Insulin Prescribing for Patients With Diabetes During the COVID-19 Pandemic in the US

**DOI:** 10.1001/jamanetworkopen.2021.32607

**Published:** 2021-11-03

**Authors:** Ismaeel Yunusa, Bryan L. Love, Chao Cai, Tessa Hastings, Claiborne E. Reeder, Elizabeth W. Blake, Cynthia Phillips

**Affiliations:** 1 Center for Outcomes Research and Evaluation, University of South Carolina College of Pharmacy, Columbia; 2 Department of Clinical Pharmacy and Outcomes Sciences, University of South Carolina College of Pharmacy, Columbia; 3 Kennedy Pharmacy Innovation Center, University of South Carolina College of Pharmacy, Columbia

## Abstract

This cross-sectional study compares the number of insulin prescriptions filled before and during the COVID-19 pandemic.

## Introduction

For the approximately 7 million US individuals with diabetes who rely on insulin,^1^ the number of insulin prescriptions rose steadily in the decade before the COVID-19 pandemic.^2^ Reduced access to medical care and disruptions in supply related to the pandemic may have decreased access to insulin,^3^ with possible adverse consequences for diabetes control.^4^ Conversely, emergency Medicaid expansions that increased capacity for telemedicine services during the pandemic may have been associated with improved access to treatment.^5^ Therefore, this study aimed to assess whether changes to insulin prescription claims occurred during the COVID-19 pandemic.

## Methods

In this cross-sectional study, we used pharmacy claims from a 10% random sample of patients with diabetes and at least 1 insulin claim from the US IQVIA Longitudinal Prescription Claims (LRx) data, a longitudinal dataset of patient prescriptions based on retail pharmacy data, to examine trends in prescription claims for insulin products. We counted the number of new and refill weekly insulin prescriptions between January 2019 and October 2020, except for the week surrounding the pandemic declaration (March 16, 2020) and weeks with national holidays because of expected outliers in prescription volume. Using an interrupted time series design with segmented regression analysis, we examined whether the pandemic changed the number of new and existing insulin prescription fills in adults and children. The regression model estimated weekly baseline prescription counts and their 95% CIs in 2019, the trend before the COVID-19 pandemic, and changes in trend immediately after the pandemic declaration and during the pandemic. Statistical significance was determined when the 95% CI did not include 0. All analyses were conducted using Stata statistical software, version 15.1 (StataCorp, LLC). The University of South Carolina’s Institutional Review Board deemed the study exempt from review and waived the requirement for informed consent because we used deidentified data. This report followed the Strengthening the Reporting of Observational Studies in Epidemiology (STROBE) reporting guideline.

## Results

A total of 285 343 individuals met the study criteria; the mean (SD) age was 56.6 (18.5) years, and 148 110 (51.9%) were women. The weekly number of insulin prescriptions increased steadily before the pandemic (Figure). The baseline average count of all existing insulin prescriptions excluding weeks with major holidays in the first week of 2019 was estimated to be 17 037.5 (95% CI, 16 728.7-17 346.4). The estimated number of insulin prescriptions increased significantly every week before the pandemic by 11.0 (95% CI, 2.8-19.3). In the first week of the pandemic, the mean number of prescriptions decreased by −395.6 (95% CI, −933.5 to 142.4) per week, followed by a significant decrease of −55.3 (95% CI, −78.6 to −32.0) prescriptions per week during the pandemic vs before the pandemic. In addition, we found that the pandemic was associated with a significant decrease in the estimated mean number of weekly insulin prescriptions in the adult subgroup (−54.2; −76.5 to −31.8), but not in the pediatric and new prescription subgroups (Table).

**Figure.**
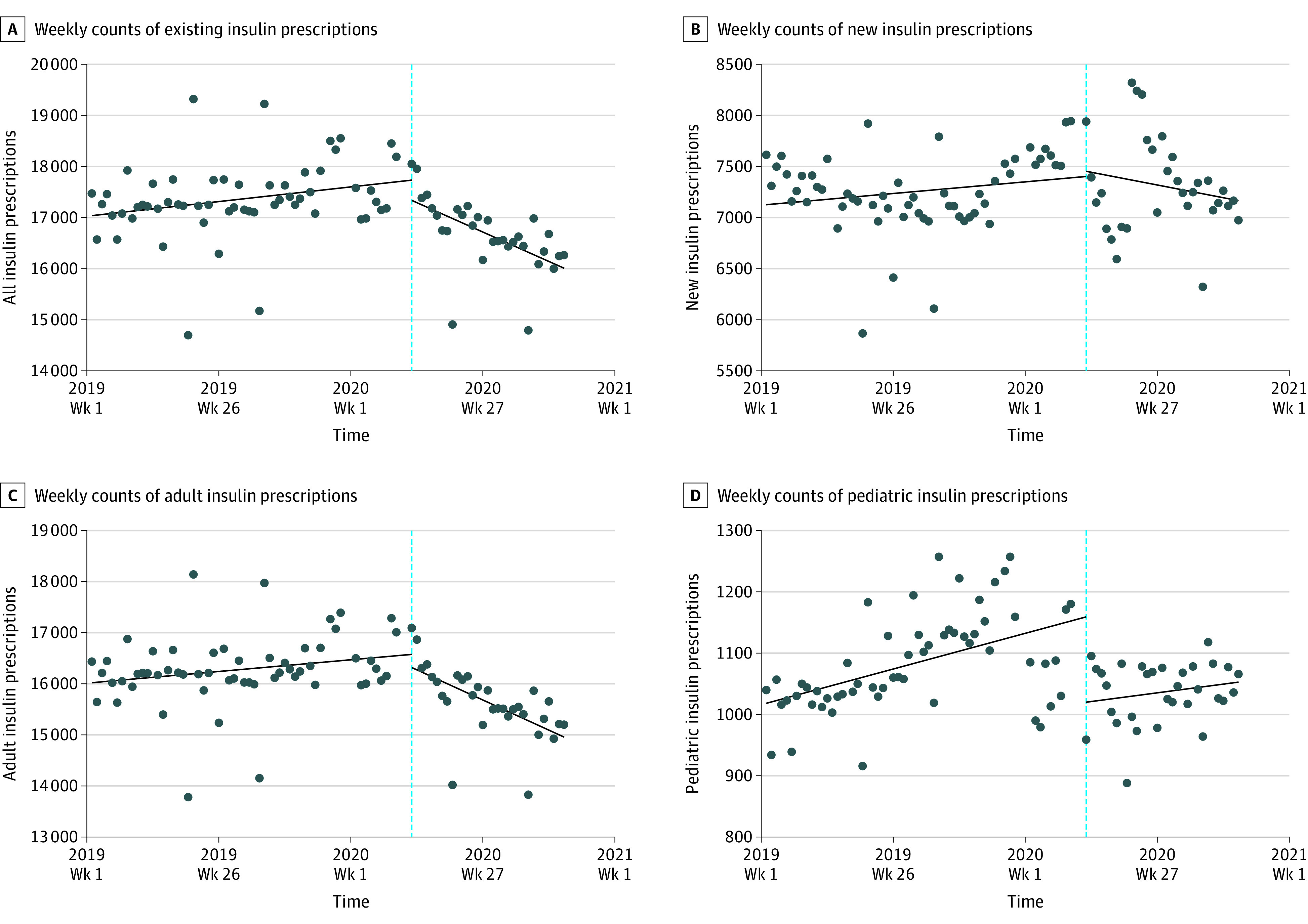
Trends in the Number of Insulin Prescriptions in the US From 2019 to 2020 The vertical dotted lines indicate the start of the COVID-19 pandemic on March 16, 2020. Interrupted time series analysis was performed with regression and Newey-West standard errors lag(0).

**Table.** Changes in Weekly Insulin Prescription Fill Trends in the US Before and During the COVID-19 Pandemic

Prescription group and measure^a^	Estimated mean No. of prescriptions (95% CI)^b^
**All existing **	
Baseline count	17 037.5 (16 728.7 to 17 346.3)
Prepandemic trend	11.0 (2.8 to 19.3)
Pandemic week immediate change	−395.6 (−933.5 to 142.4)
Trend change during pandemic	−55.3 (−78.6 to −32.0)
**All new**	
Baseline count	7126.1 (6946.8 to 7305.4)
Prepandemic trend	4.4 (−0.5 to 9.3)
Pandemic week immediate change	50.8 (−346.5 to 448.1)
Trend change during pandemic	−14.0 (−30.2 to 2.3)
**Adult^c^**	
Baseline count	16 019.4 (15 729.6 to 16 309.2)
Prepandemic trend	8.8 (1.3 to 16.3)
Pandemic week immediate change	−256.4 (−759.3 to 246.5)
Trend change during pandemic	−54.2 (−76.5 to −31.8)
**Pediatric^c^**	
Baseline count	1018.1 (990.3 to 1046.0)
Prepandemic trend	2.2 (1.1 to 3.3)
Pandemic week immediate change	−139.2 (−202.5 to −75.9)
Trend change during pandemic	−1.2 (−3.4 to 1.1)

^a^ Trend change in the number of insulin prescriptions during pandemic is based on data from January 2019 through October 2020.

^b^ Based on average weekly prescription fills. Where (95% CI) did not include 0 indicates statistical significance.

^c^ Adult and pediatric prescriptions were based on all existing prescriptions during the study period.

## Discussion

This study found a considerable decrease in the average number of weekly insulin prescription fills during the COVID-19 pandemic. Reduced contact with prescribing clinicians during the pandemic, rationing, previous stockpiling, or loss of insurance could explain the decline.^6^ The lack of substantial decline in pediatric patients’ prescriptions can be explained by the fact that insulin use in pediatrics is more likely for type 1 diabetes. In addition, relatively smaller sample size may have limited statistical power to detect a difference in prescribing trends for an age group for which insulin is the mainstay of treatment. Although telehealth services may have reduced care disruption, it may not have led to more insulin prescription fills. The limitations of this study include its retrospective design, its descriptive nature, and a lack of data to adjust for an association of mortality with the reduced number of insulin fills. However, we believe that these findings contribute to a better understanding of the association of the pandemic with insulin treatment for diabetes. Future studies should examine whether the pandemic was associated with adverse health outcomes associated with suboptimal insulin treatment.
